# Case Report: Angiographic Remodeling of a Chronic Total Occlusion Four Years After Extraplaque Wiring: Implications for Procedural Reattempt

**DOI:** 10.1093/ehjcr/ytag226

**Published:** 2026-03-27

**Authors:** Hassan Saleh, Jaikirshan Khatri

**Affiliations:** NewYork Presbyterian/Weill Cornell Medical Center, 525 E 68th St, New York, NY 10065, USA; NewYork Presbyterian/Weill Cornell Medical Center, 525 E 68th St, New York, NY 10065, USA

**Keywords:** Chronic total occlusion, CTO, PCI, Investment procedure, Case report

## Abstract

**Background:**

An investment procedure during chronic total occlusion (CTO) percutaneous coronary intervention (PCI) involves intentional alteration of the proximal cap with the aim of facilitating subsequent CTO PCI. Extraplaque wiring may alter the CTO segment without modification of the proximal cap; however, whether such alterations meaningfully improve future CTO PCI success is uncertain.

**Case Summary:**

A 71-year-old gentleman with a CTO of the second obtuse marginal and Canadian Cardiovascular Society (CCS) class III symptoms underwent an initial CTO PCI attempt that was aborted after extraplaque wiring without balloon dilation. Four years later, repeat angiography demonstrated shortening of the CTO segment. During reattempt, an initial polymer-jacketed wire tracked extraplaque, and successful crossing was achieved using parallel wiring, followed by stent implantation with restoration of TIMI 3 flow.

**Discussion:**

Interval change was observed within the CTO segment years after prior extraplaque instrumentation. The mechanism underlying this change is uncertain and may reflect lesion evolution, extraplaque manipulation, or a combination of factors. Importantly, despite shortening of the occluded segment, this did not simplify crossing at reattempt, which remained technically complex. These findings suggest that shortening of the CTO segment alone should not be assumed to confer procedural advantage and highlight the need for further systematic evaluation of staged CTO strategies.

Learning points

- Interval change in the CTO body may be observed years after prior extraplaque instrumentation, with some changes being more favourable than others for facilitating a reattempt at CTO PCI.
- Shortening of the CTO segment after extraplaque wiring may be due to fenestrations created between the extraplaque and intraplaque space.

## Introduction

CTO PCI remains technically challenging despite advances in equipment and techniques, with contemporary success rates of 85%–90% among high-volume operators.^1,2^ Modern crossing algorithms emphasize dual angiography and anatomical assessment to guide crossing strategy, with most operators starting with antegrade wire escalation.^3^ A recent concept in CTO PCI is an investment procedure—a staged attempt in which the operator modifies the proximal cap, extraplaque space, or plaque morphology without achieving final distal recanalization with the goal of facilitating future success.^4,5^ We present a case demonstrating interval shortening of a CTO segment 4 years after a failed CTO PCI attempt limited to extraplaque wiring, highlighting questions regarding lesion evolution and staged CTO strategies.

## Summary figure

**Figure:**
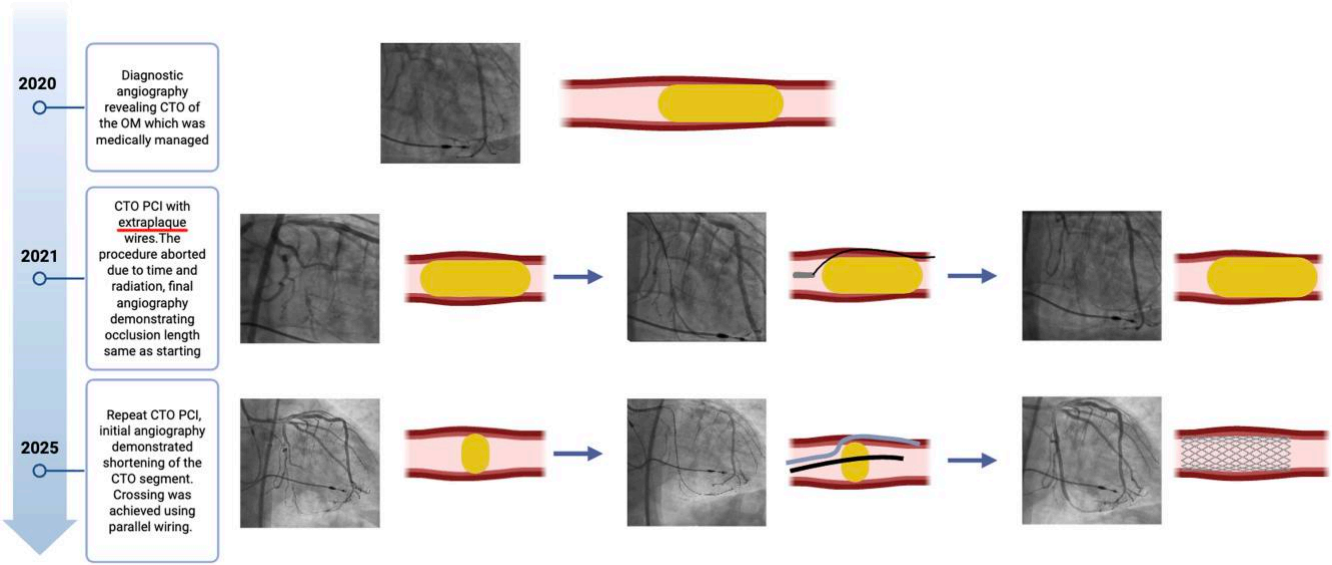
Top panel: Total occlusion of the obtuse marginal 5 years prior, managed medically. Middle panel: Four years prior—initial angiography (left), extraplaque wiring during attempted CTO PCI (middle), and final angiography after aborted procedure demonstrating persistent TIMI 0 flow (right). Bottom panel: Present day—initial angiography demonstrating apparent shortening of the occluded segment (left), parallel wiring with distal true lumen entry (middle), and final angiography following drug-eluting stent implantation with restoration of TIMI 3 flow (right).

## History of presentation

A 71-year-old gentleman with a CTO of the second obtuse marginal (OM2) and intolerance to ranolazine was referred for CTO PCI. Five months prior, he was in the clinic and reported chest pressure and dyspnoea while hiking that progressed to symptoms with moderate activity while on metoprolol succinate 100 mg daily and amlodipine 10 mg daily. Isosorbide mononitrate 30 mg daily was started. He had some improvement with nitrates, but symptoms progressed to CCS Class III over the course of the next 5 months. Coronary computed tomography angiography (CCTA) was performed, revealing total occlusion of the OM2, a calcium score of 52 in the circumflex, and no other significant stenoses.

## Medical history

The patient had a history of single-vessel coronary artery disease, hypertension, diabetes mellitus type II, hyperlipidaemia, and complete heart block for which a dual-chamber pacemaker was placed 5 years prior.

## Investigations

### Initial CTO PCI

**Figure 1:**
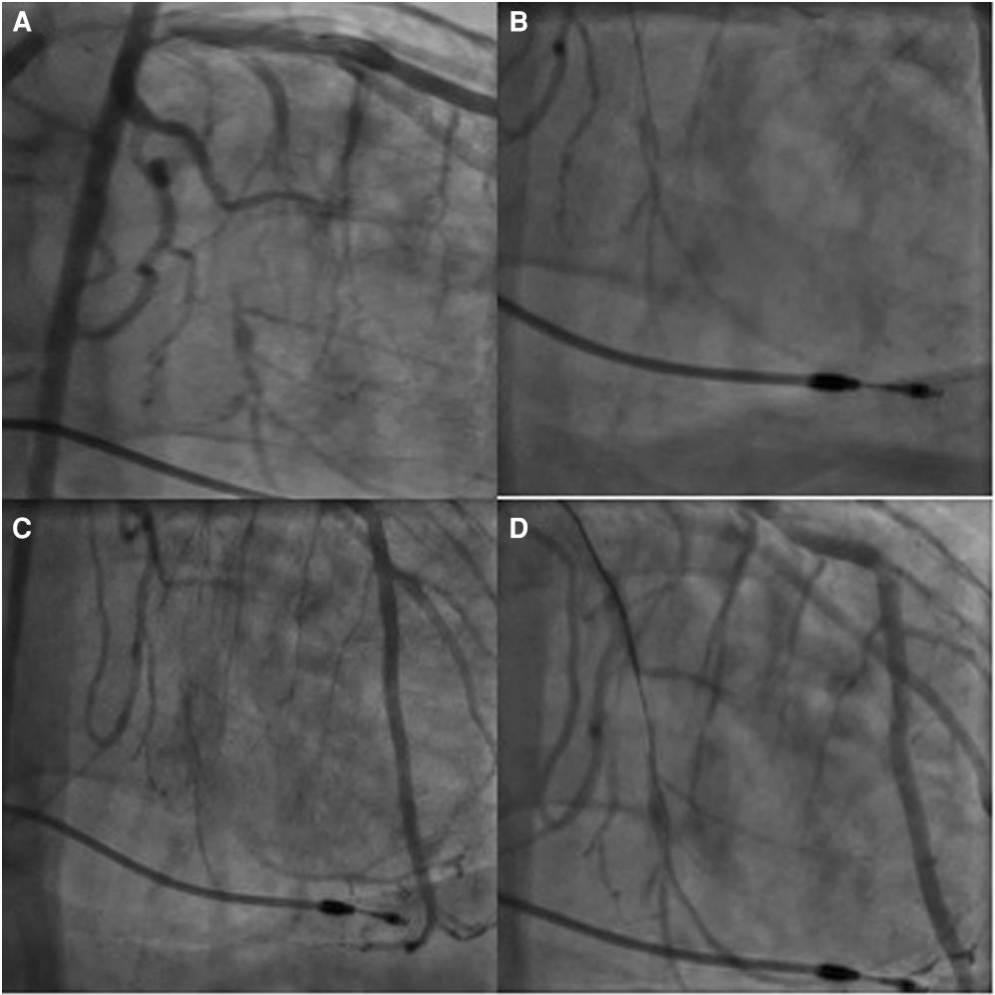
Clockwise from top left, *(A)* initial angiography at first CTO PCI attempt, *(B)* extraplaque wire, *(C)* extraplaque wire with dissections, and *(D)* final angiography with likely haematoma in the collateralized segment.

Five years prior to this presentation, the patient underwent coronary angiography, revealing CTO of the OM2, which was initially managed medically but then underwent attempted CTO PCI 1 year later due to progression of symptoms. During that CTO PCI, antegrade wiring using a Fielder XT and intravascular ultrasound (IVUS)-guided puncture using a Gaia Next 2 were attempted but was unsuccessful, and the procedure was aborted (*Figure 1*). Final angiography demonstrated thrombolysis in myocardial infarction (TIMI) flow grade 0 (see Supplementary material online, *Video S1*).

### Second CTO PCI

One month following CCTA, the patient was brought for CTO PCI. Dual access was obtained using a 5/6 French Glidesheath Slender (Terumo, Somerset, NJ) in the right radial artery and an 8 French × 45 cm Super Flex Arrow (Teleflex, Morrisville, NC) in the right common femoral artery (CFA). Intravenous heparin was administered to achieve an activated clotting time (ACT) > 300 s. An 8 French EBU 3.75 guide (Medtronic, Minneapolis, MN) was used to cannulate the left main. Angiography was then performed (see Supplementary material online, *Video S2*), revealing antegrade flow in the OM2 CTO that was not present at the end of the previous CTO PCI attempt, apparently shortening the length of the CTO segment (see Supplementary material online, *Video S1*).

**Figure 2:**
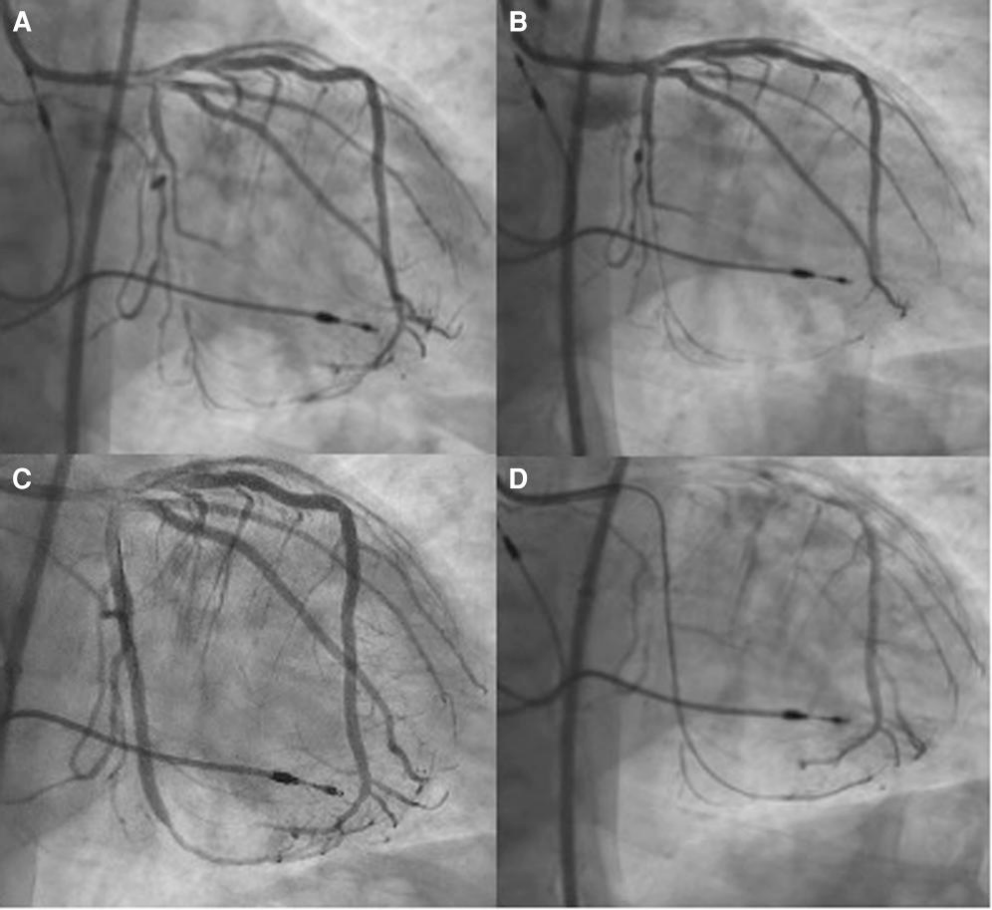
Clockwise from top left, *(A)* initial angiography and second CTO PCI, *(B)* Fielder XT-A in the extraplaque space, *(C)* extralaque Fielder XT-A and intraplaque Gaia Next 1, and *(D)* final angiography.

A Sion Blue (Asahi Intecc, Seto, Japan) and Turnpike LP (Teleflex, Morrisville, NC) were advanced to the proximal cap of the OM2 CTO. The Sion Blue was exchanged for a Fielder XT-A (Asahi Intecc, Seto, Japan), which then entered the extra plaque space (*Figure 2*). The Turnpike LP was removed using a TrapIT (Interventional Medical Device Solutions, Roden, Netherlands), and a Sion Blue loaded in the Turnpike LP was advanced to the proximal cap of the CTO. The Sion Blue was exchanged for a Gaia Next 1 (Asahi Intecc, Seto, Japan), which, despite multiple attempts, would not enter the proximal cap.

An Nhancer Rx (Interventional Medical Device Solutions, Roden, Netherlands) was loaded on the Fielder XT-A, which also would not advance beyond the proximal cap. Using a semicompliant 1.5 × 20 Takeru (Terumo, Somerset, NJ) on the Fielder XT-A, the proximal cap was dilated. The Nhancer Rx was loaded on the Fielder XT-A, which crossed into the proximal cap. The Gaia Next 1 was placed over the wire port of the Nhancer Rx, which traversed the CTO segment and remained true lumen with wire position confirmed in multiple views (*Figure 2*). The Fielder XT-A and the Nhancer Rx were removed. The Gaia Next 1 was exchanged for a Sion blue using the Turnpike LP and a TrapIT.

The lesion was prepared with a semi-compliant 2.0 × 30 Euphora (Medtronic, Minneapolis, Minnesota), followed by IVUS with OPTICROSS HD IVUS (Boston Scientific, Marlborough, MA), revealing a distal reference of 3.0 mm and a proximal reference of 3.8 mm. A 3.0 × 38 Xience Skypoint (Abbott, Abbott Park, IL) was advanced to the target segment and deployed at 12 atmospheres (atm). Post-dilation was performed with a 3.0 × 20 non-compliant Euphora for the distal and middle thirds of the stent at 18 atm and a 4.0 × 8 non-compliant Euphora for the proximal third at 18 atm. IVUS was repeated, revealing adequate stent apposition and expansion. Final angiography demonstrated TIMI Grade 3 Flow (see Supplementary material online, *Video S3*).

## Management

He was observed and discharged the next day on dual antiplatelet therapy.

## Discussion

This case offers a real-world demonstration that CTO morphology may evolve years after an unsuccessful CTO PCI. A lesion that remained TIMI 0 after a failed attempt, which was not a deliberate investment strategy, without the passage of balloons, dedicated re-entry devices, or true lumen entry, shortened on repeat angiography. The mechanism underlying this change is uncertain and may reflect natural lesion evolution, prior subintimal manipulation, or a combination of factors. Given the limited characterization of the CTO before angiography at reattempt, a causal relationship cannot be established.

However, a strategy of an investment procedure in technically complex CTOs has been proposed to facilitate success in a future attempt. In this case, it should be noted that while the CTO segment shortened, it did not facilitate success. This case raises the question of what types of manipulation result in a successful investment procedure, as well as what happens to a CTO years after manipulation. Current literature suggests that modification be achieved using balloon angioplasty or delivery of dedicated re-entry devices, with attempts at wire re-entry, with optimal timing currently thought to be greater than 30 days.^4–7^

Despite shortening of the occlusion, the second procedure remained technically complex. Thus, shortening of the CTO segment did not translate into a simplified procedural strategy. This observation highlights that angiographic alteration of a CTO segment should not be presumed to confer procedural advantage. While the concept of staged modification continues to be explored, systematic evaluation is required to determine what interval morphological changes influence reattempt outcomes.

## Conclusions

This case illustrates interval shortening of a CTO segment years after prior extraplaque instrumentation. The mechanistic basis of these changes remains uncertain, and apparent shortening did not result in simplified crossing at reattempt. These findings underscore the need for further understanding of morphological changes following manipulation of a CTO.

## Supplementary Material

ytag226_Supplementary_Data

## Data Availability

The data underlying this article are available in the article and its online Supplementary material

## Lead author biography

Hassan Saleh is a current CHIP fellow at New York Presbyterian/Weill Cornell Medical Center. Prior to this, he completed his Interventional Cardiology training at the University of Louisville and General Cardiology training at Corewell Health/Michigan State University in Grand Rapids, MI.

## Notes

**Consent:** Informed consent was obtained from the patient for the publication of this case report and accompnanying images in accordance with COPE (Committee on Publication Ethic) guidelines. All identifying information has been removed to ensure patient confidentiality.
