# Supplementary material for: Case Report: Angiographic Remodeling of a Chronic Total Occlusion Four Years After Extraplaque Wiring: Implications for Procedural Reattempt
Source: Eur Heart J Case Rep. 2026 Mar 27;10(4):ytag226. doi: 10.1093/ehjcr/ytag226 (PMC13082229; doi:10.1093/ehjcr/ytag226)
Supplement: ytag226_Supplementary_Data [file ytag226_supplementary_data.zip › Supplementary video captions.docx]

Video 1. Final angiography following CTO PCI attempt four years prior.

Video 2. Initial angiography at start of second CTO, note the shortening of the CTO segment relative to final angiography in figure 1.

Video 3. Final angiography following second CTO PCI attempt.
